# Supplementary material for: The causal relationship between the human gut microbiota and pyogenic arthritis: a Mendelian randomization study
Source: Front Cell Infect Microbiol. 2024 Nov 26;14:1452480. doi: 10.3389/fcimb.2024.1452480 (PMC11629706; doi:10.3389/fcimb.2024.1452480)
Supplement: Supplementary file 1 [file DataSheet1.zip › mendelian test/ebi-a-GCST90027809.csv_leave_one_out.pdf]

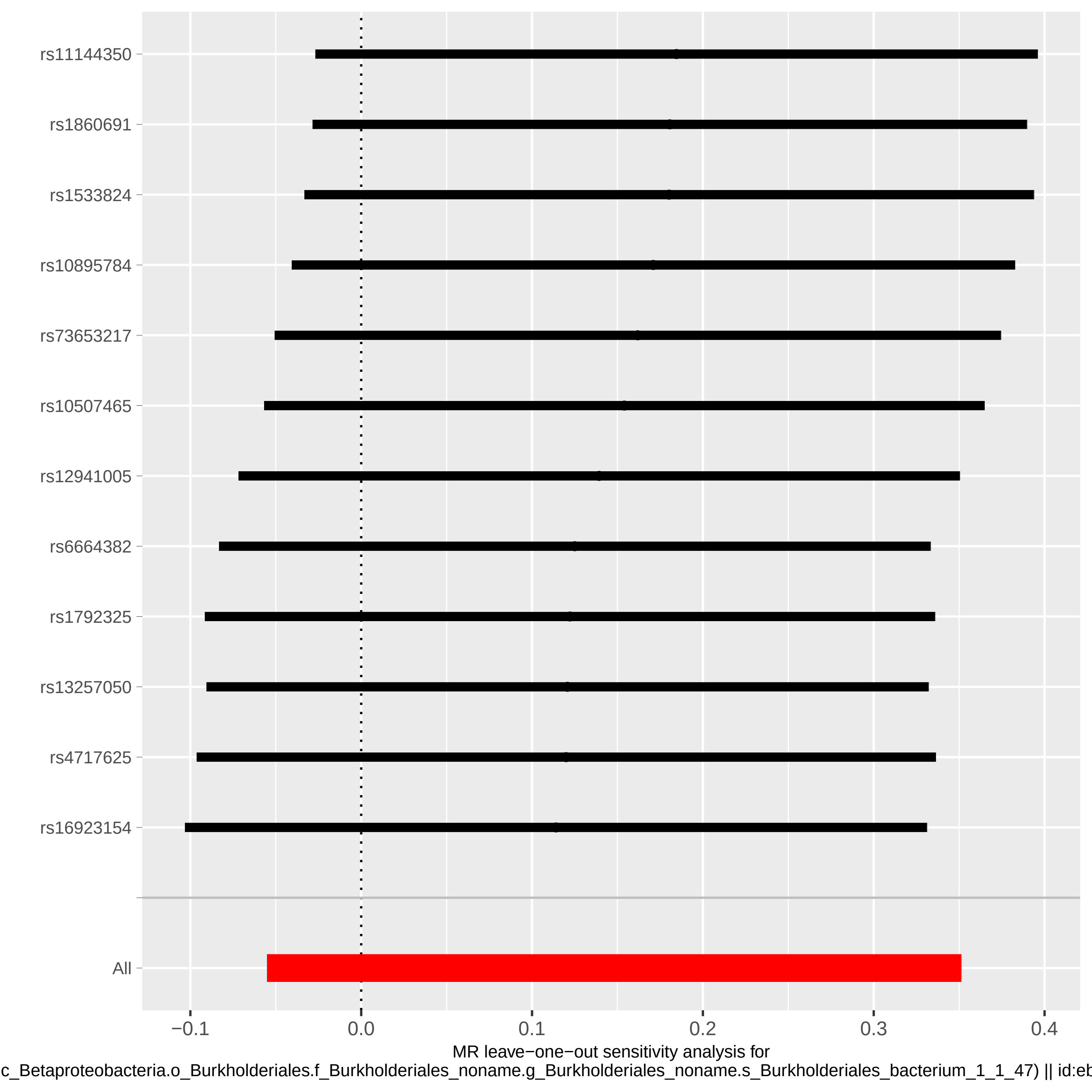
rs11144350
rs1860691
rs1533824
rs10895784
rs73653217
rs10507465
rs12941005
rs6664382
rs1792325
rs13257050
rs4717625
rs16923154
All
−0.1
0.0
0.1
0.2
0.3
0.4
MR leave−one−out sensitivity analysis for
c_Betaproteobacteria.o_Burkholderiales.f_Burkholderiales_noname.g_Burkholderiales_noname.s_Burkholderiales_bacterium_1_1_47) || id:eb
